# Urine High-Sensitive Troponin T—Novel Biomarker of Myocardial Damage in Children

**DOI:** 10.31083/j.rcm2405147

**Published:** 2023-05-18

**Authors:** Matija Bakoš, Duje Braovac, Ana-Meyra Potkonjak, Tomo Svaguša, Tomislav Ćaleta, Daniel Dilber, Dorotea Bartoniček, Boris Filipović-Grčić, Slobodan Galić, Ana Lončar Vrančić, Željka Vogrinc, Željko Đurić, Mislav Planinc, Milivoj Novak, Toni Matić

**Affiliations:** ^1^Department of Pediatrics, University Hospital Centre Zagreb, 10000 Zagreb, Croatia; ^2^Department of Gynecology and Obstetrics, Sestre milosrdnice University Hospital Centre, 10000 Zagreb, Croatia; ^3^Department of Cardiology, Dubrava University Hospital, 10000 Zagreb, Croatia; ^4^School of Medicine, University of Zagreb,10000 Zagreb, Croatia; ^5^Department of Laboratory Diagnostics, University Hospital Centre Zagreb, 10000 Zagreb, Croatia; ^6^Department of Cardiac Surgery, University Hospital Centre Zagreb, 10000 Zagreb, Croatia

**Keywords:** troponin T, urine, cardiac surgery, ventricular septal defect, bidirectional cavopulmonary connection

## Abstract

**Background:**

The use of high-sensitive cardiac troponin T (hsTnT) in urine as a marker of cardiac damage in children has not yet been reported. Elimination of cardiac troponins is dependent on renal function; persistently increased serum hsTnT concentrations were observed among individuals with impaired renal function. The aim of this study was to investigate serum and urine hsTnT levels and its correlation in infants and children younger than 24 months of age after cardiac surgery.

**Methods:**

This study was conducted on 90 infants and children under 24 months of age who were divided into three groups. The experimental group consisted of patients with intracardiac surgery of ventricular septal defect (VSD), first control group consisted of infants with extracardiac formation of bidirectional cavopulmonary connection (BCPC), and the second control group consisted of healthy children. Troponin T values ​​were determined in serum and urine at five time points: the first sample was taken on the day before cardiac surgery (measure 0) and the other four samples were taken after the surgery; immediately after (measure 1), on the first (measure 2), third (measure 3), and fifth postoperative day (measure 5). The first morning urine was sampled for determining the troponin T in the control group of healthy infants.

**Results:**

A positive correlation between troponin T values in serum and urine was found. Urine hsTnT measured preoperatively in children undergoing BCPC surgery was higher (median 7.3 [IQR 6.6–13.3] ng/L) compared to children undergoing VSD surgery (median 6.5 [IQR 4.4–8.9] ng/L) as well as to healthy population (median 5.5 [IQR 5.1–6.7] ng/L). After logarithmic transformation, there was no statistically significant difference in urine hsTnT concentration between the groups at any point of measurement preoperatively or postoperatively. Statistically significant negative correlation was found between serum and urine hsTnT concentrations and glomerular filtration rate estimated by creatinine clearance. Patients who underwent surgical repair of VSD had significantly higher concentrations of troponin T in serum on the first three postoperative measurements compared to those who had BCPC surgery.

**Conclusions:**

According to the results of this study, renal function after cardiac surgery appears to have a major effect on the urinary hsTnT concentrations, and we cannot conclude that this is an appropriate marker for the assessment of postoperative myocardial damage in children. Nevertheless, more research is needed to reach a better understanding of the final elimination of cardiac troponins in children.

## 1. Introduction

The use of high-sensitive cardiac troponin T (hsTnT) in urine as a marker of myocardial damage in children has not yet been reported. A recent publication in adult patients suffering from acute myocardial damage showed the possibility of detecting hsTnT in urine [1]. Elimination of cardiac troponins is dependent on renal function; persistently increased serum hsTnT concentrations were observed among individuals with impaired renal function [2, 3]. Both renal impairment and myocardial damage in children can be observed after congenital heart defects (CHD) surgeries. CHD represents one of the most common congenital malformations, accounting for almost one-third (28%) of all congenital anomalies [4]. According to numerous studies, the prevalence of CHD is approximately 9 per 1000 live births worldwide [5, 6]. The most common heart defect in the Croatian national study, as well as in other studies, is ventricular septal defect (VSD) with an incidence of 34.6% of all heart defects [5, 6]. Markers of myocardial damage investigated in children include creatine kinase (CK), creatine kinase MB (CK-MB), myoglobin, cardiac troponin T and I in blood. Cardiac troponins are located inside heart myocytes and can enter the bloodstream in the case of myocyte damage. Myocardial injury is a significant cause of mortality and morbidity after cardiac surgery in children. For the assessment of myocardial injury after cardiac surgery in children, both troponins are used [7, 8, 9]. Serum elevation of both cardiac troponins was found to be a good predictor of complications and adverse clinical events after surgery [10]. Children show a postoperative increase in serum troponin levels after intracardiac surgery, in contrast to children after extracardiac surgery (e.g., bidirectional cavopulmonary connection (BCPC)) [8, 11]. In this study hsTnT was used instead of high-sensitive troponin I (hsTnI) because hsTnT is routinely used in our institution.

The aim of this study was to investigate the dynamics of serum and urine hsTnT levels in infants and children younger than 24 months of age after cardiac surgery of ventricular septal defect and after BCPC.

## 2. Materials and Methods

This study was conducted in 90 infants and children below 24 months of age (58 male, 32 female) at the University Hospital Centre (UHC) Zagreb, Zagreb, Croatia between November 2014 and August 2021. The experimental group consisted of patients with VSD who underwent cardiac surgery (group 1). Two control groups were involved in the research: the first control group consisted of infants after BCPC surgery (group 2) and the second control group consisted of healthy children who were examined at our Department of Pediatric Cardiology, in whom the absence of a CHD was confirmed by echocardiography (group 3).

Demographic, clinical, duration of stay in pediatric intensive care unit (PICU), duration of hospital stay, duration of mechanical ventilation, mortality, morbidity, and surgical-related (time of cardiopulmonary bypass, time of aortic cross-clamp) data were collected.

Information on healthy subjects was collected on pediatric examination. The subjects were excluded from the study if the examination revealed other known acute and/or chronic diseases. In children operated for CHD, data were obtained mainly from medical history data and through direct contact with the parents of the children. Prior to enrollment in the study, parental written informed consent for all patients was obtained.

Patients aged up to 24 months of age, who underwent surgical closure of VSD or BCPC surgery were considered eligible for the study. Healthy infants included in the study were those without CHD and other acute or chronic diseases. Associated syndrome of known etiology and incomplete data were the reasons for exclusion from the study.

This research was approved by the Ethics Committee of the UHC Zagreb (number 02/21 AG).

Troponin T values ​​were determined from serum and urine at five time points: the first sample was taken on the day before cardiac surgery (measure 0) and the other four samples were taken after the surgery; immediately after (measure 1), on the first (measure 2), third (measure 3), and fifth postoperative day (measure 5). Serum was the sample of choice because patients also required protein electrophoresis and immunoglobulin testing for which serum is the preferred sample. To avoid additional blood draw serum was selected. The first morning urine was sampled for determining the troponin T in the control group of healthy infants. Urine was collected from a urinary catheter or urine collection bag. Immediately after withdrawing blood samples from each individual subject, serum was separated from the blood, and was stored at the temperature of –20 °C. After the samples of all subjects were collected, the concentration of troponin T in serum and urine was determined in UHC Zagreb, Clinical Institute for Laboratory Diagnostics on the Roche Cobas 6000 CEE device (Roche Diagnostics International, Rotkreuz, Switzerland). Troponin T was measured in serum and urine by the electrochemiluminescence method using Roche Troponin T high sensitivity (hs) STAT reagent (Roche Diagnostics GmbH, Mannheim, Germany). Roche Troponin T high sensitivity (hs) STAT immunoassay is intended for the *in vitro* quantitative determination of hsTnT in human serum and plasma. The linearity of the Roche hsTnT STAT test is 3–10,000 ng/L (Limit of Blank 2 ng/L, Limit of Detection 3 ng/L). The 99th percentile for troponin T determination with Roche hsTnT STAT immunoassay is 14 ng/L (95 % confidence interval 12.7–24.9 ng/L with ≤10% co-efficient of variation (CV)) [12]. In troponin T analysis, the first 50 μL of sample is incubated for 9 minutes with biotinylated monoclonal anti-troponin T specific antibody, monoclonal anti-troponin T specific antibody labeled with a ruthenium complex and streptavidin coated microparticles. All reaction substances form a sandwich complex. Reaction mixture is then transferred into the measuring cell where the microparticles are magnetically bound to the surface of the electrode and all unbound material is washed. Voltage is applied to the electrode which induces chemiluminescent emission which is measured by a photomultiplier. Results are read from the calibration curve. Detailed information is available on the product data sheet (Roche Troponin T (hs) STAT, REF 05092728 190). Creatinine concentration in serum and urine was measured spectrophotometrically using the Roche reagent Diagnostics Creatinine Plus 2 (Roche Diagnostics GmbH, Mannheim, Germany). The extent of kidney damage was determined before and after the surgery by estimating the glomerular filtration rate (eGFR) using the Schwartz “bedside” formula which employs serum creatinine [eGFR (mL/min/1.73 $m^{2}$ = 0.413 × height (cm)/serum creatinine level (mg/dL))] [13]. Because urinary concentration of any biomarker is dependent on the urinary flow rate as well as on the biomarker excretion rate, urinary biomarker concentrations are commonly expressed as a ratio to the urinary creatinine concentration [14, 15]. The same was done in this study: troponin T in urine was divided with creatinine in urine (tropTU/UCr).

### Statistical Analysis

For all variables analyzed in the study descriptive statistics were done. For all statistical tests, a significance level of 5% (*p *< 0.05) was considered statistically significant unless otherwise stated. Variables - Troponin T in serum and in urine and tropTU/UCr were logarithmically transformed (ln transformation) due to the non-normality of the distribution. After logarithmic transformation, the distribution was normal. Differences between groups at point zero measurement (before surgery) for weight, body surface area (BSA), troponin T in serum and in urine, and creatinine were examined using univariate analysis of variance (ANOVA). For each day (day on admission to the PICU, 1st, 3rd, and 5th postoperative day) independently, the differences of logarithmic transformation of troponin T in urine (lnTropTU), in serum (lnTropTS), as well as the tropTU/UCr (ln_TropTU/UCr) between groups were examined using a univariate ANOVA. Tukey’s post hoc analysis was used to identify the cause of the difference if the results of the ANOVA test indicated that there was a statistically significant difference between the groups. Repeated measures ANOVA (RM ANOVA) tested the differences between lnTropTS, lnTropTU and ln_TropTU/UCr between the groups during the analyzed period (measurements, days) and the interaction between groups and days. If the measurement component turned out to be statistically significant, the contrast of each measurement with the last, 5th measurement, was tested. Also, the correlation between creatinine clearance and lnTropT levels in serum and urine for each analyzed day using correlation analysis was investigated, as well as the correlation between lnTropTS and lnTroptU. All statistical analyzes were performed in the statistical package SAS9.4 (SAS Institute Inc, Cary, NC, USA).

## 3. Results

### 3.1 Demographic and Clinical Data by Groups

#### 3.1.1 Patients with VSD

A total of 49 children with VSD were included in the study. The mean age was 9.5 (standard deviation [SD] 5.1) months (min 1.5–max 23.5), and the mean weight was 7.1 [2.0] kg (3.5–12.8). The mean BSA was 0.37 [0.075] $m^{2}$ (0.22–0.58). The mean length of stay in the PICU was 5.5 [4.6] days (2–28), while the mean in-hospital stay was 21.9 [14.5] days (8–67). The mean number of hours on mechanical ventilation after VSD surgery was 39.4 [58.0] hours (0–306). The mean duration of cardiopulmonary bypass (CPB) during the operation was 106 [32.0] minutes (54–215), and the duration of aortic clamping (AOX) was 62.4 [22.0] minutes (26–124). The median value of troponin T before surgery in serum and in urine was 16.5 [IQR 8.4–35.2] ng/L and 6.5 [IQR 4.4–8.9] ng/L, respectively. The median value of tropTU/UCr before surgery was 2.7 [IQR 0.8–9.2] ng/μmol.

#### 3.1.2 Patients with BCPC Surgery

A total of 18 patients who underwent BCPC procedure were included in the study. The mean age at surgery was 4.8 [1.0] months (3–6.5), and the mean weight was 6.2 [0.9] kg (4.4–7.4). The mean BSA was 0.33 [0.034] $m^{2}$ (0.26–0.38). The mean length of stay at the PICU in the postoperative period was 10.2 [6.9] days (4–31), while the mean in-hospital stay was 36.6 [26.9] days (16–133). The mean duration of mechanical ventilation was 50.9 [97.0] hours (0–407). Six infants (33%) were weaned from mechanical ventilation in the operating room, six of them in the PICU in the first 24 hours after surgery, while in three infants mechanical ventilation lasted for more than 100 hours. The mean duration of CPB was 107 [40.8] minutes (48–169). The median value of troponin T before surgery in serum and in urine was 20.9 [IQR 16.3–36] ng/L and 7.3 [IQR 6.6–13.3] ng/L, respectively. The median value of tropTU/UCr before surgery was 3.3 [IQR 2.3–12.5] ng/μmol.

#### 3.1.3 Healthy Controls

A total of 23 healthy infants whose urine was successfully stored were examined through the pediatric cardiology ward. The mean BSA was 0.39 [0.063] $m^{2}$ (0.29–0.53). The mean age was 7.5 [3.9] months (1.5–19.5), and the mean weight was 8.2 [1.7] kg (5.4–12.5). The median value of troponin T in urine was 5.5 [IQR 5.1–6.7] ng/L, while the median value of tropTU/UCr was 3.4 [IQR 1.8–6.2] ng/μmol.

### 3.2 Measured Values—Preoperative Measurements

Although higher values were observed in the BCPC group than in both healthy and VSD groups, no statistically significant difference in lnTropTU was found (Table 1). Similarly, no difference was found in ln_TropTU/UCr values between the groups. Almost equal values of lnTropTS were observed in the VSD and BCPC groups.

**Table 1.** **Results of the analysis of variance in preoperative measurements by groups**.

Variables	Groups	N	Mean	St.Dev.	Statistics	*p*-value
lnTropTU	1	46	1.82	0.55	F = 2.02	0.1398
	2	14	2.09	0.63		
	3	23	1.78	0.28		
ln_TropTU/UCr	1	39	0.98	1.28	F = 1.01	0.3707
	2	14	1.49	1.23		
	3	23	1.19	0.88		
lnTropTS	1	46	2.9	0.96	F = 2.59	0.1129
	2	15	3.23	0.64		

Abbreviations: N, number of patients; St.Dev., standard deviation; 1, patients after cardiac surgery of ventricular septal defect; 2, patients after cardiac surgery of bidirectional cavopulmonary connection; 3, healthy controls; lnTropTU, logarithmic transformation of troponin T in urine; ln_TropTU/UCr, logarithmic transformation of ratio of troponin T in urine and urine creatinine; lnTropTS, logarithmic transformation of troponin T in serum.

### 3.3 Repeated Measure ANOVA (RM ANOVA) 

**Fig. 1.**
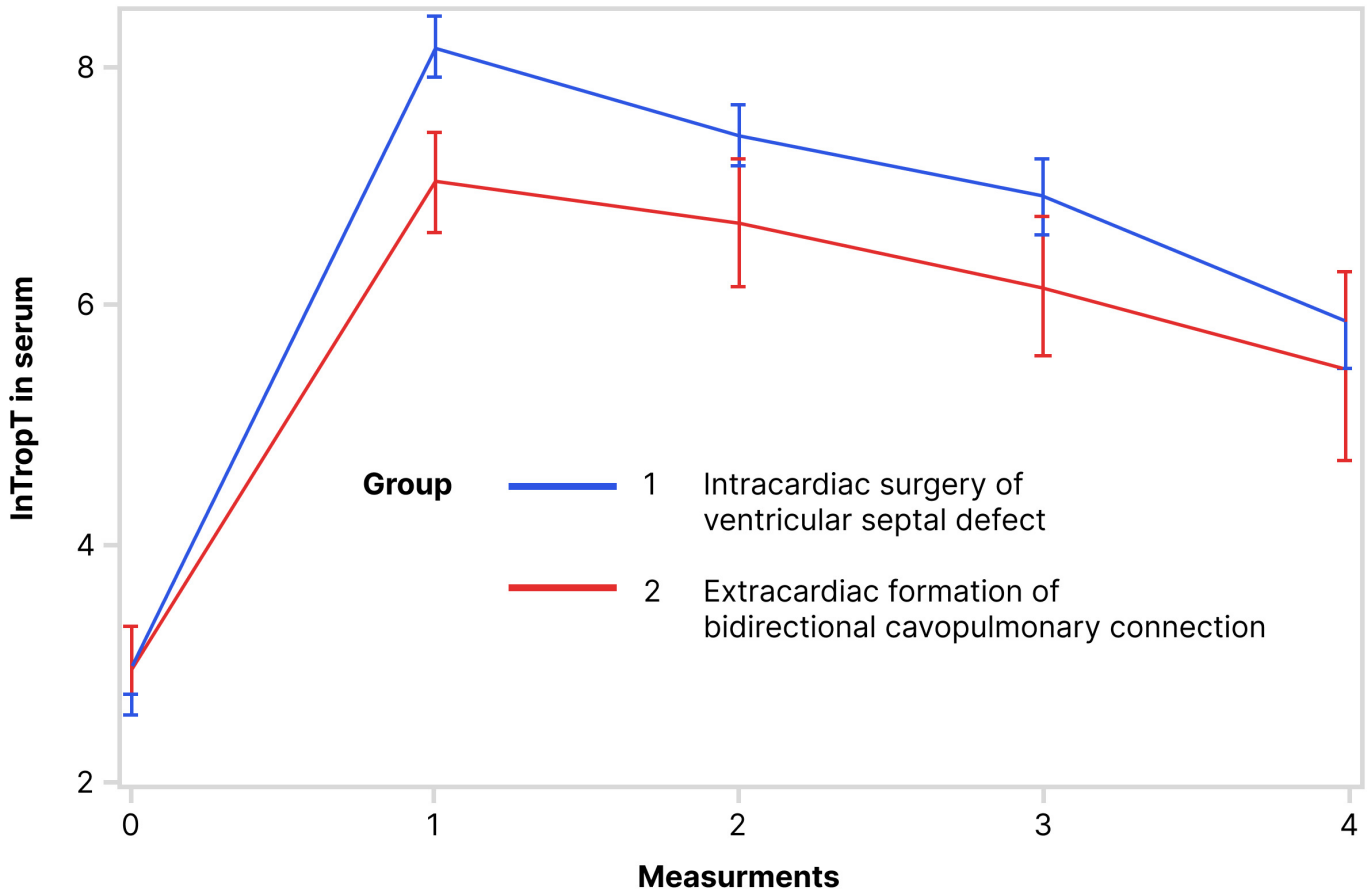
**Trend of lnTropT in serum during observed period (RM ANOVA): RM ANOVA test showed that there is a statistically significant difference in serum lnTropT between the following groups**. Abbreviation: lnTropT, logarithmic transformation of troponin T.

**Fig. 2.**
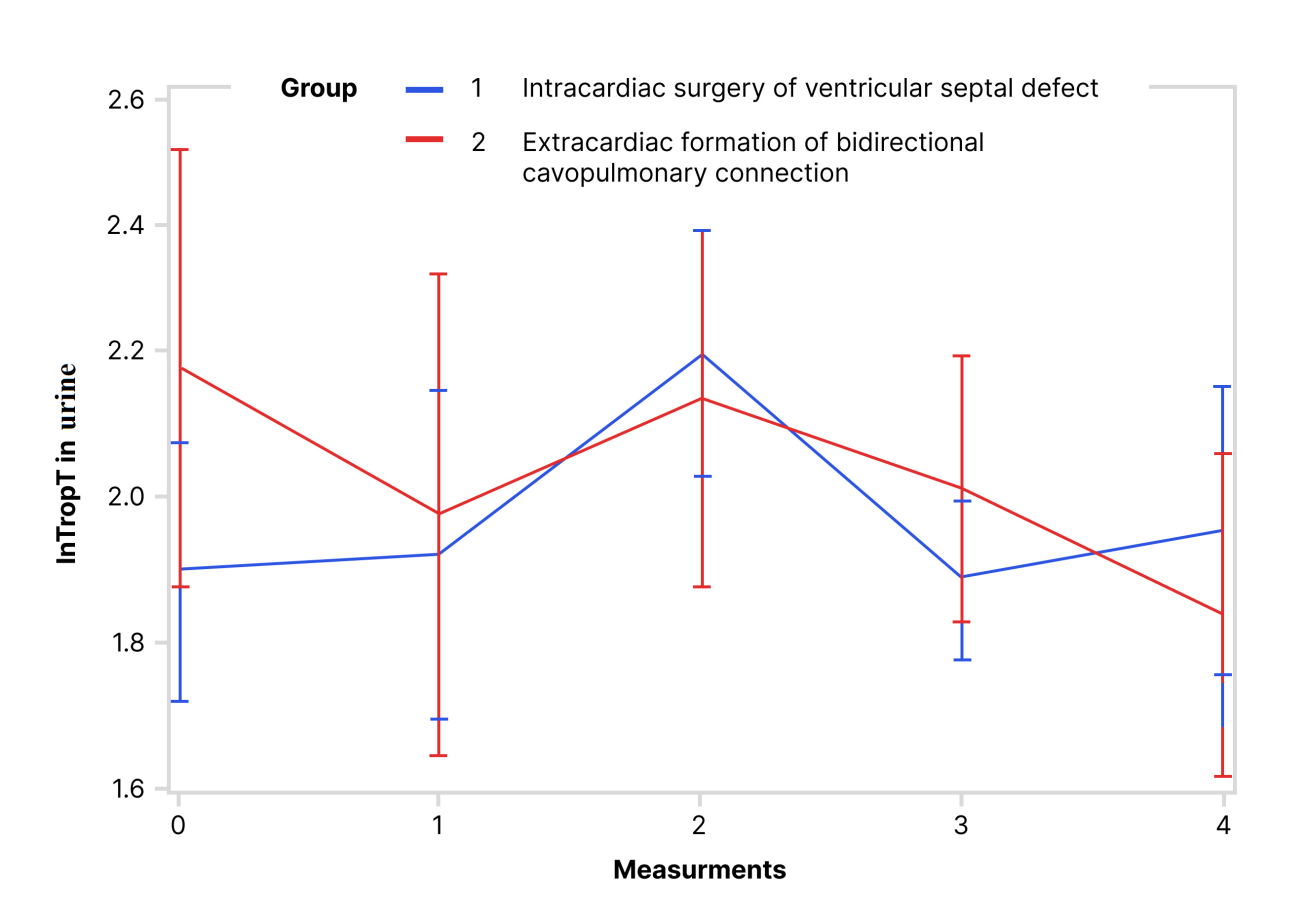
**Trend of lnTropT in urine during the observed period (RM ANOVA): RM ANOVA test showed that there is no statistically significant difference in urine lnTropT between the following groups**. Abbreviation: lnTropT, logarithmic transformation of troponin T.

**Fig. 3.**
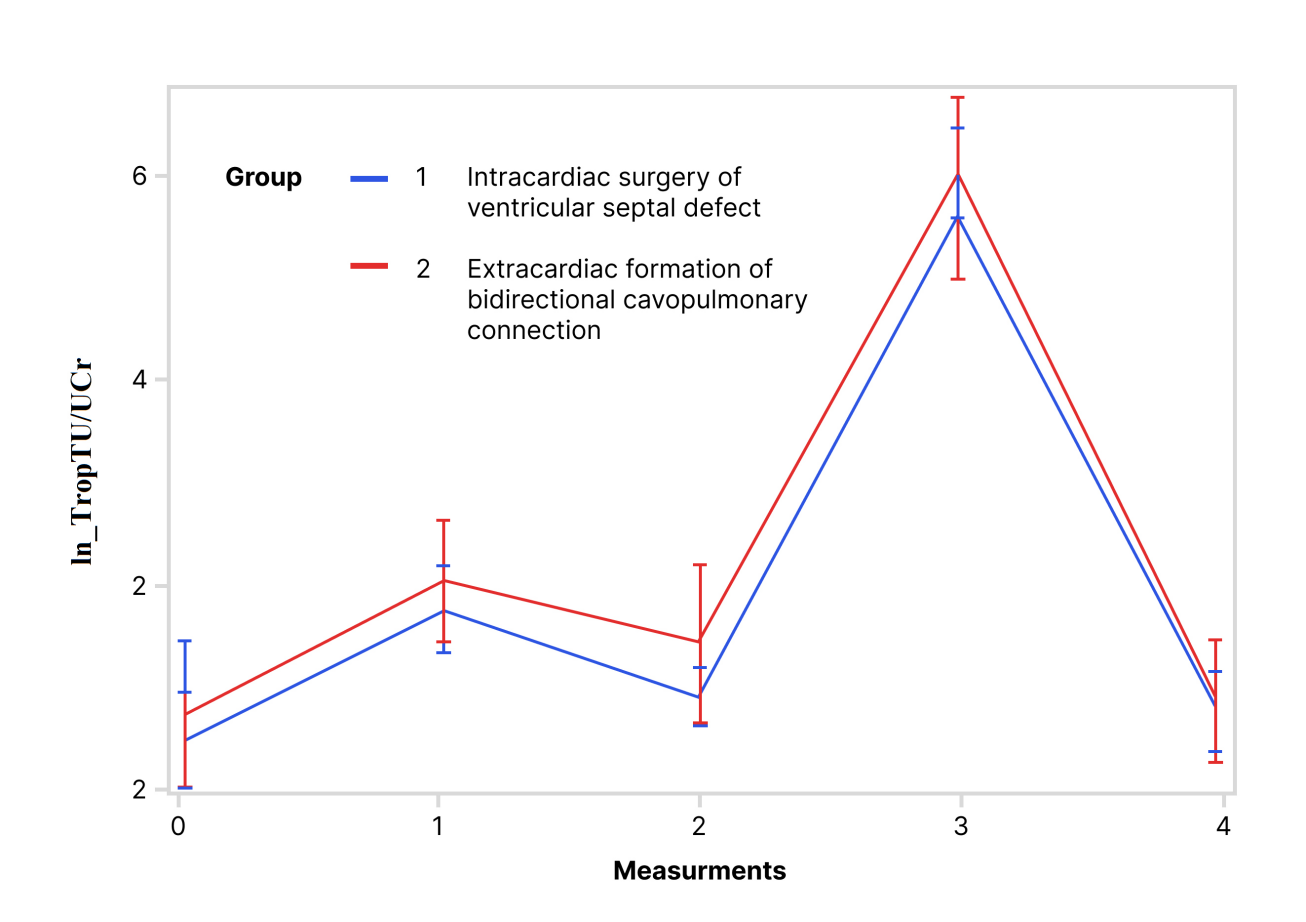
**Trend of ln_TropTU/UCr during the observed period (RM ANOVA): RM ANOVA test showed that there is no statistically significant difference in ln_TropTU/UCr between the following groups**. Abbreviation: ln_TropTU/UCr, logarithmic transformation of ratio of troponin T in urine and urine creatinine.

Values of lnTropTS, lnTropTU and ln_TropTU/UCr during observed period are shown in Figs. 1,2,3, separately. RM ANOVA test showed that there is a statistically significant difference between the groups (VSD and BCPC) for lnTropTS (Fig. 1), while for lnTropTU (Fig. 2) and for ln_TropTU/UCr (Fig. 3) this difference was not statistically significant (Group in Table 2). If we observe those values considering different measurements (Time in Table 2), the difference was found for lnTropTS and ln_TropTU/UCr values (for lnTropT measurements 0, 1, 2, and 3 (Fig. 1); for ln_TropTU/UCr measurements 1 and 3 (Fig. 3), both *p *< 0.0001), while for lnTropTU during the same period no difference was found (*p* = 0.0524) (Table 2) Interactions (time × group in Table 2) between the analyzed values during the period (measurements) and groups (VSD and BCPC) were statistically significantly different only for troponin T in the serum (Fig. 1), interfering that lnTropT values in serum do not have the same trend in the observed period for both groups. The similar effect of interaction between analyzed period and groups was not statistically different for lnTropTU and ln_TropTU/UCr. This means that both lnTropTU and ln_TropTU/UCr have similar trends in the observed period in the two groups (Figs. 2,3).

**Table 2.** **Results of the repeated measure analysis of variance for lnTropTS, lnTropTU and ln_TropTU/UCr**.

Source of variability	df	lnTropTS		lnTropTU		ln_TropTU/UCr	
		F value	*p*> F	F value	*p*> F	F value	*p*> F
Group	1	5.11	0.0292	0.27	0.6033	0.89	0.3521
Time	4	394.23	< ${\mathrm{0.0001}}^{1}$	2.63	${\mathrm{0.0524}}^{1}$	150.36	<0.0001
Time × Group	4	6.37	${\mathrm{0.0031}}^{1}$	1.40	${\mathrm{0.2455}}^{1}$	0.46	0.7675
Sphericity Tests	9	$x^{2}$ = 106.27	<0.0001	$x^{2}$ = 24.89	0.0031	$x^{2}$ = 9.912	0.3576

Note: ^1^ adjusted with Greenhouse-Geisser Epsilon. Abbreviations: lnTropTS, logarithmic transformation of troponin T in serum; lnTropTU, logarithmic transformation of troponin T in urine; ln_TropTU/UCr, logarithmic transformation of ratio of troponin T in urine and urine creatinine; df, degree of freedom; $x^{2}$, chi-squared test.

The univariate ANOVA was used separately for each day of the observed period (Table 3). The results showed that for lnTropTS there was statistically significant difference between the VSD and BCPC groups on the 1st, 2nd, and 3rd measurement, while in the 5th, this difference was no longer statistically significant. For lnTropTU and ln_TropTU/UCr these differences between the groups per measurements exist, but are not statistically significant (Table 3, Figs. 1,2,3).

**Table 3.** **Results of univariate analysis of variance by analyzed days for lnTropT in serum and urine and ln_tropTU/UCr between groups 1 (VSD) and 2 (BCPC)**.

Time (measurement)	Source of variability	lnTropTS		lnTropTU		ln_TropTU/UCr	
		F value	*p >* F	F value	*p *> F	F value	*p >* F
1	Group (1, 2)	23.38	<0.0001	0.08	0.7841	0.55	0.4617
2	Group (1, 2)	8.17	0.0067	0.15	0.7044	2.63	0.1130
3	Group (1, 2)	5.89	0.0197	1.57	0.2172	0.12	0.7348
5	Group (1, 2)	0.98	0.3286	0.47	0.4977	0.07	0.7877

Abbreviations: lnTropTS, logarithmic transformation of troponin T in serum; lnTropTU, logarithmic transformation of troponin T in urine; ln_TropTU/UCr, logarithmic transformation of ratio of troponin T in urine and urine creatinine.

### 3.4 Pearson Correlation Coefficients

A positive correlation was found between lnTropTS and lnTropTU in the first, second and third measurement, in contrast to zero, and fifth measurement where this correlation was very weak (Table 4). Creatinine clearance showed a negative correlation with lnTropT both in urine and in serum in the first three measurements. This correlation was more negative for urine values. This means that in patients with higher creatinine clearance lower values of troponin T were observed in both serum and urine. In the measurement before surgery, as well as in the first and second postoperative measurement, this negative correlation is statistically significant (Table 4). Positive correlation was found between lnTropT values in urine and serum and duration of CPB as well as with duration of AOX (only in the first group) (Table 4). Duration of mechanical ventilation positively correlated with both lnTropT values in urine and the serum, in most of the measurements (Table 4).

**Table 4.** **Correlations between logarithmic transformation of troponin T values (serum and urine) and analyzed variables**.

Pearson Correlation Coefficients (PC) and significance (S)											
Meas.		0		1		2		3		5	
		lnTropTU	lnTropTS	lnTropTU	lnTropTS	lnTropTU	lnTropTS	lnTropTU	lnTropTS	lnTropTU	lnTropTS
lnTropTS by meas.	PC	0.022		0.455		0.377		0.309		0.161	
	S	0.865		0.0002		0.002		0.016		0.245	
CrCl by meas.	PC	–0.350	–0.241	–0.240	–0.127	0.390	0.220	–0.010	–0.120	–0.040	–0.080
	S	0.006	0.061	0.054	0.347	0.001	0.080	0.912	0.333	0.779	0.573
CPB	PC	0.087	0.478	0.421	0.429	0.306	0.534	0.279	0.449	0.164	0.412
	S	0.509	<0.0001	0.0005	0.0004	0.014	<0.0001	0.029	0.0002	0.231	0.002
PICU stay	PC	0.144	0.186	0.266	0.003	0.259	0.137	0.284	0.165	–0.022	0.269
	S	0.272	0.152	0.152	0.981	0.039	0.283	0.026	0.201	0.874	0.049
Hosp. stay	PC	0.016	0.432	0.185	–0.049	0.097	0.146	0.078	0.141	–0.007	0.205
	S	0.904	0.0005	0.143	0.696	0.443	0.255	0.549	0.275	0.957	0.137
Mech. vent.	PC	0.257	0.307	0.496	0.228	0.524	0.321	0.398	0.383	–0.025	0.343
	S	0.047	0.016	<0.0001	0.070	<0.0001	0.010	0.002	0.002	0.856	0.011
AOX (group 1)	PC	0.064	0.448	0.443	0.493	0.282	0.547	0.312	0.414	0.152	0.358
	S	0.670	0.002	0.002	0.0004	0.052	<0.0001	0.037	0.004	0.361	0.029

Abbreviations: Meas., measurement; lnTropTS, logarithmic transformation of troponin T in serum; lnTropTU, logarithmic transformation of troponin T in urine; CrCl, creatinine clearance; CPB, cardiopulmonary bypass; PICU, pediatric intensive care unit; hosp. stay, hospital stay; mech. vent., mechanical ventilation; AOX, aortic cross clamping.

## 4. Discussion

A positive correlation between troponin T values in serum and urine was found. There was no statistically significant difference in urine troponin T concentration between the groups in any point of measurement preoperatively or postoperatively. Statistically significant negative correlation was found between serum and urine troponin T concentrations and glomerular filtration rate (GFR) estimated by creatinine clearance. Patients who underwent surgical repair of VSD had significantly higher concentrations of troponin T in serum on the first three postoperative measurements compared to those who had BCPC surgery.

Troponin is an intracellular protein that is important for the regulation of muscle contraction [16]. Three types of troponins are known (troponin I, T and C) [17]. Troponins I and T are cardiac troponins that are routinely determined in the blood. They are highly specific markers of myocardial damage, and their increased concentration in the blood is a sign of heart damage. The concentration of both markers in the blood increases two to three hours after the heart damage, and it reaches its maximum value 24 hours after and remains elevated up to 8 days [18, 19]. Troponin T is a larger molecule than troponin I, with molecular mass of 43 kilodaltons (kDa) [2]. After it appears in the blood, the troponin molecule is broken down into smaller segments so that the entire troponin molecule is not present after 12 hours [20]. The molecular mass of the largest degradation segment is about 20 kDa (fragment of troponin T) [2]. Fridén *et al*. [3] hypothesized in their study that more than a half of the measured hsTnT with a molecular weight below 17 kDa have a relatively free passage over the glomerular membrane. Similarly, they found that at very high levels, significant role in clearance of troponin T molecule has extrarenal mechanism including mononuclear phagocyte system [3]. Numerous cardiac and extracardiac conditions can cause an increase of troponins in children and adults. Those causes can be divided into three groups including primary heart diseases and lesions (i.e., cardiomyopathies, cardiac arrhythmias, heart surgery, etc.), non-cardiac and systemic pathologies that affect the myocardium (i.e., sepsis, chronic diseases, pulmonary embolism, systemic hypoxia, COVID-19, etc.), and interference in the preanalytical stage and intra-laboratory problems (i.e., hemolysis, lipemia, ictericity, sample collection error) [21]. Latter was observed in patients with skeletal myopathies where hsTnT was chronically elevated, probably due to cross-reaction of the troponin T immunoassay with skeletal muscle troponin isoform [22, 23]. Nowadays, high-sensitivity immunoassays are recommended as a gold standard laboratory method to detect myocardial injury both in adults [24] and in children [25]. A significant advantage of the fifth generation of hsTnT is a lower detection limit, which is ten to hundred times higher than the detection limits of third and fourth generations [21]. Moreover, the sensitivity of hsTnT was shown to be so high that it opened the possibility of non-invasive detection (urine, oral fluid) [21]. Several studies have reported the presence of troponin molecules in urine and the kidneys are being considered as the main route of excretion [1, 26, 27, 28, 29]. However, to the best of our knowledge, this study is the first to examine troponin T in urine as a marker of myocardial damage in the pediatric population. Recently, Streng *et al*. [1] showed that hsTnT can be present in the urine in adult patients with acute myocardial infarction. In their study, the fifth generation of hsTnT immunoassay (by Roche Diagnostics) was used, as well as in our study. The measured values of urine hsTnT were significantly higher than those measured in healthy controls [1]. The authors discussed the uncertainty of immunoreactivity because of the urinary matrix. In recent times, urine has become a more popular biofluid used in studies due to its non-invasiveness, easiness to collect, and the possibility of repeating the measurements. The interpretation of results obtained from urine samples could be challenging due to interference of results with salts and urea, broad pH spectrum, and low protein concentrations [30]. Furthermore, there is a high intraindividual variability of urine metabolites concentration over a short period of time. Avoidance of these variabilities is mostly achieved by expressing the metabolite levels in relation to urinary creatinine which was also done in our study [14, 15].

One of the hypotheses of the present study was to test whether urine hsTnT can be used as a noninvasive tool for the assessment of myocardial damage in children.

The finding of this study that the patients who underwent surgical repair of VSD have significantly higher concentrations of troponin T in serum on the first three postoperative measurements compared to those who had BCPC surgery, was expected. During the surgery of heart defects, cardiac myocytes are damaged, and cardioselective enzymes are released [31, 32]. In the pediatric population, blood troponins show a good correlation with the severity of myocardial damage after cardiac surgery [31, 32]. The greater the myocyte damage is, the higher is the concentration of troponin in the blood, which explains the higher concentration of troponin in patients after intracardiac VSD repair compared to those after BCPC surgery found in this study. Many authors found that higher blood hsTnT concentration is related to the complexity of the surgical procedure, duration of CPB and AOX [31, 32]. The concentration of hsTnT can also be predictive of the time of hospitalization in the PICU [31, 32, 33, 34]. Similar results were confirmed in this study. Preoperative values of serum hsTnT were not increased in this study. An increase in preoperative cardiac troponins is rare in patients with stable heart disease, and if present, it negatively affects the outcome of the surgery [7, 35].

This research has confirmed that the level of hsTnT in urine of children with congenital heart disease can be determined, but it did not show significant difference between intracardiac and extracardiac groups of cardiosurgical patients, as did the serum hsTnT. We also found that urine hsTnT measured preoperatively in children undergoing BCPC surgery (median 7.3 [IQR 6.6–13.3] ng/L) was higher compared to that in the healthy population (median 5.5 [IQR 5.1–6.7] ng/L). After dividing these values to urinary creatinine, the values were approximately the same (median 3.3 [IQR 2.3–12.5] in BCPC group vs. median 3.4 [IQR 1.8–6.2] ng/mmol in healthy population). However, in both cases after logarithmic transformation of values, no statistically significant difference was found. The reason could be the relatively small sample size. Two similar studies, except for investigating troponin I molecule, were conducted by Chen *et al*. [29] and Pervan *et al*. [28]. Chen *et al*. [29] demonstrated that a single measurement of troponin I in fresh urine sample may be an acceptable marker for predicting cardiovascular events in patients with diabetes mellitus. Pervan *et al*. [28] found that troponin I in first morning urine was higher in adult patients with hypertension than that in healthy population. Recently, Westreich *et al*. [36] showed that urine hsTnT excretion in patients with myocardial infarction (MI) was significantly increased compared to healthy individuals. They also found that patients with MI and kidney disease had higher values of hsTnT in urine compared to patients with MI and without kidney disease [36]. Similar findings were also observed in this study, where the negative correlation of creatinine clearance and hsTnT was stronger in urine than in serum. The kidneys are vital in metabolism and clearance of small peptides and proteins which are partially filtered through the filtration barrier and then enter the proximal tubule where they are reabsorbed [37]. Numerous biomarkers, including cardiac ones, are processed by the kidney in a similar fashion [38]. The metabolism and excretion of troponins are affected by changes in eGFR and multiple studies have shown that patients with reduced eGFR have an elevated cardiac troponin T level in blood [38, 39, 40]. Few other studies also found the same inverse relationship between urine troponin T and eGFR [26, 41]. The explanation could be that the kidney injury is predominantly intrinsic and leads to restricted tubular reabsorption which contributes to the higher level of troponin T in urine [26].

The limitation of the study, apart from a relatively small sample size, is that the sample used for analysis of hsTnT was plasma, and not both plasma and serum. Although the fifth generation of hsTnT has been validated for use from both plasma and serum in adults [42], the same study was not performed on children.

## 5. Conclusions

According to the results of this study renal function after cardiac surgery appears to have a major effect on the urinary hsTnT concentrations, and we cannot conclude that this is an appropriate marker for the assessment of postoperative myocardial damage in children. Nevertheless, more research is needed to reach a better understanding of the final elimination of hsTnT in children.

## Data Availability

All data generated or analyzed during this study are included in this published article.
